# Fauna Europaea: Helminths (Animal Parasitic)

**DOI:** 10.3897/BDJ.2.e1060

**Published:** 2014-09-17

**Authors:** David I. Gibson, Rodney A. Bray, David Hunt, Boyko B. Georgiev, Tomaš Scholz, Philip D. Harris, Tor A. Bakke, Teresa Pojmanska, Katarzyna Niewiadomska, Aneta Kostadinova, Vasyl Tkach, Odile Bain, Marie-Claude Durette-Desset, Lynda Gibbons, František Moravec, Annie Petter, Zlatka M. Dimitrova, Kurt Buchmann, E. Tellervo Valtonen, Yde de Jong

**Affiliations:** † Natural History Museum, London, United Kingdom; ‡ CABI Europe-UK, Egham, United Kingdom; § Central Laboratory of General Ecology (BAS), Sofia, Bulgaria; | Biology Centre (CAS), České Budějovice, Czech Republic; ¶ University of Oslo, Oslo, Norway; # W. Stefański Institute of Parasitology (PAS), Warsaw, Poland; †† University of North Dakota, Grand Forks, United States of America; ‡‡ Museum National d'Histoire Naturelle, Paris, France; §§ Royal Veterinary College, London, United Kingdom; || Thracian University, Stara Zagora, Bulgaria; ¶¶ University of Copenhagen, Copenhagen, Denmark; ## University of Jyväskylä, Jyväskylä, Finland; ††† University of Eastern Finland, Joensuu, Finland; ‡‡‡ University of Amsterdam - Faculty of Science, Amsterdam, Netherlands

**Keywords:** Biodiversity Informatics, Fauna Europaea, Taxonomic indexing, Zoology, Biodiversity, Taxonomy, Helminth, Acanthocephala, Cestoda, Monogenea, Trematoda, Nematoda, Parasite

## Abstract

*Fauna Europaea* provides a public web-service with an index of scientific names (including important synonyms) of all living European land and freshwater animals, their geographical distribution at country level (up to the Urals, excluding the Caucasus region), and some additional information. The *Fauna Europaea* project covers about 230,000 taxonomic names, including 130,000 accepted species and 14,000 accepted subspecies, which is much more than the originally projected number of 100,000 species. This represents a huge effort by more than 400 contributing specialists throughout Europe and is a unique (standard) reference suitable for many users in science, government, industry, nature conservation and education.

Helminths parasitic in animals represent a large assemblage of worms, representing three phyla, with more than 200 families and almost 4,000 species of parasites from all major vertebrate and many invertebrate groups. A general introduction is given for each of the major groups of parasitic worms, i.e. the Acanthocephala, Monogenea, Trematoda (Aspidogastrea and Digenea), Cestoda and Nematoda. Basic information for each group includes its size, host-range, distribution, morphological features, life-cycle, classification, identification and recent key-works. Tabulations include a complete list of families dealt with, the number of species in each and the name of the specialist responsible for data acquisition, a list of additional specialists who helped with particular groups, and a list of higher taxa dealt with down to the family level. A compilation of useful references is appended.

## Introduction

The European Commission published the European Community Biodiversity Strategy, providing a framework for the development of Community policies and instruments in order to comply with the Convention on Biological Diversity. This Strategy recognises the current incomplete state of knowledge at all levels concerning biodiversity, which is a constraint on the successful implementation of the Convention. The Fauna Europaea contributes to this Strategy by supporting one of the main themes: to identify and catalogue the components of European biodiversity into a database in order to serve as a basic tool for science and conservation policies.

With regard to biodiversity in Europe, both science and policy depend on a knowledge of its components. The assessment of biodiversity, monitoring changes, sustainable exploitation of biodiversity and much legislative work depend upon a validated overview of taxonomic biodiversity. Towards this end, the Fauna Europaea plays a major role, providing a web-based information infrastructure with an index of scientific names (including important synonyms) of all living European land and freshwater animals, their geographical distribution at country level and some additional useful information. In this sense, the Fauna Europaea database provides a unique reference for many user-groups, such as scientists, governments, industries, conservation communities and educational programmes.

The Fauna Europaea started in 2000 as an EC-FP5 four-year project, delivering its first release in 2004. After 13 years of steady progress, in order to improve the dissemination of Fauna Europaea results and to increase the general awareness of work done by the Fauna Europaea contributors, novel e-Publishing tools have been used to prepare data papers for all 58 major taxonomic groups. This contribution represents the first publication of the Fauna Europaea Helminths (Animal Parasitic) data sector as a BDJ data paper.

## General description

### Purpose

The Fauna Europaea is a database of the scientific names and distribution of all living, currently known multicellular European land and freshwater animal species assembled by a large network of experts using advanced electronic tools for data collation and validation routines.

The 'Helminths (animal parasitic)' is one of the 58 major Fauna Europaea taxonomic groups, covering 3,986 species. The data were acquired and checked by a network of 19 specialists (Tables 1, 2).

### Additional information

 

**HELMITNTHS**

The animal parasitic helminths (‘parasitic worms’) dealt with in this section include members of three phyla, the Acanthocephala (‘thorny-headed worms’ or 'spiny-headed worms'), Platyhelminthes (‘flatworms’) and Nematoda (‘roundworms’); these are usually referred to as acanthocephalans, platyhelminths and nematodes, respectively. Parasitic worms are usually parasitic at the adult stage, but many are also parasitic as larvae. Many have complex life-cycles involving the ‘definitive’ or ‘final’ host (usually a vertebrate), which harbours the adult stage, and one or more ‘intermediate hosts’ (invertebrate or vertebrate), which harbour the larval stage(s). Others have a direct life-cycle, where the definitive host is infected directly via an egg or a larval stage. Such larval stages are often encysted and survive in this state for long periods. Transmission of the parasite to the definitive host is often by ingestion with its food, or via the direct penetration by a larval stage. In nature, it is the usual condition for animals to be parasitized, so they have evolved to accommodate certain levels of infection. However, in cases where animals are kept or occur in unnaturally high concentrations, e.g. in the cases of farming, aquaculture or even man in villages or urban situations, parasite populations can build, causing them to become pathogenic. However, there are many factors, such as stress, which can cause a reduced resistance to parasites.

The classification and identification of parasitic worms have been based mainly on morphological features, although other factors, such as the host, distribution, site and life-cycle, may also be taken into consideration. In recent years, classifications based on molecular findings, which are thought to approximate closer to a true phylogenetic system, have been introduced. However, their use causes problems in identification, as classifications based on molecules and morphology are rarely totally concordant. Using a molecular classification has the disadvantage that accepted groups may not be recognised, or at least not easily recognised, using morphological criteria. Furthermore, molecular classifications are virtually always based on only an extremely small fraction of the number of taxa and individuals within the group, and consequently many taxa may be left stranded as ‘*incertae sedis*’. Therefore, although molecular evidence is considered in some recent classifications, taxonomic arrangements still tend to be based mainly on morphological and other biological criteria.

**Figure 1.**
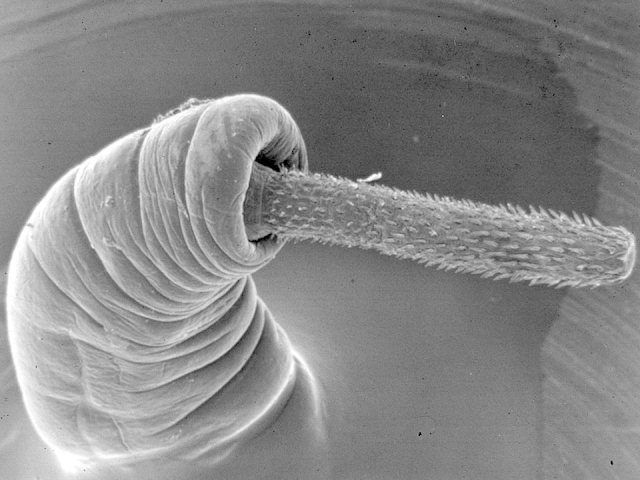
Echinorhynchid acanthocephalan.

The **ACANTHOCEPHALA** is a relatively small group of about 1,200 species. Acanthocephalans (Fig. 1) occur as intestinal parasites of a wide range of vertebrates at the adult stage, but are absent from elasmobranch fishes, and are especially prevalent in freshwater teleosts. These parasites are readily recognisable by the possession of a single large, eversible, armed proboscis. They attach using their proboscis, which penetrates the host’s intestinal wall, and resemble tapeworms in that they lack a gut and absorb nutrients from the host’s intestine through their body wall. They are dioecious and have a body-cavity which contains little but their reproductive organs. All acanthocephalans utilise arthropods as intermediate hosts (often crustaceans in the case of species parasitizing aquatic hosts and insects in cases where terrestrial mammals and birds act as final hosts), within which two larval stages occur. The second larval stage, the cystacanth, is an encysted resting stage which normally infects the vertebrate host when the arthropod host is eaten. In some cases, especially those species parasitizing piscivorous mammals and birds, a vertebrate may act as a second intermediate host in which no development occurs (a paratenic host).

In recent classifications (Monks and Richardson 2011; Amin 2013), four (three large and one small) classes are recognised. Useful taxonomic criteria at higher levels include the arrangement of lacunar canals in the syncytial body-wall, the arrangement of cement glands in the males and the nature of large nuclei in the body-wall. At lower taxonomic levels, the number and arrangement of hooks on the proboscis are the major diagnostic features. In the future, molecular studies, such as that of Weber et al. (2013), should help clarify the relationships between the different groups.

The **PLATYHELMINTHES** (flatworms) include both free-living and parasitic groups. They are bilaterally symmetrical, lack a body cavity, are composed of three main cell layers, usually lack an anus and are usually hermaphroditic. The free-living groups, referred to as the **Turbellaria**, are dealt with elsewhere, but do include a small number of parasitic or commensal forms. Parasitic platyhelminths form a group called the **Neodermata**, which comprises three distinct, divergent classes, which have in common a specialised syncytial body-covering, the tegument or neodermis, derived from mesodermal cells. The three classes are the Monogenea, the Trematoda (‘flukes’) and the Cestoda (‘tapeworms’). These groups can be so plastic in terms of their morphology and life-history that there are usually exceptions to every rule.

**Figure 2.**
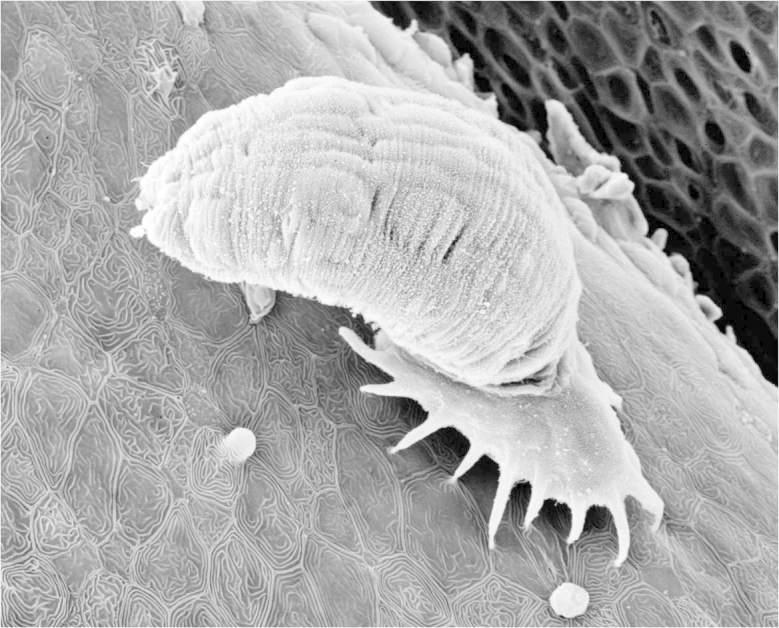
Gyrodactylid monogenean.

The **Monogenea** (also referred to as the Monogenoidea by a small number of workers, but use of this name should be avoided for several reasons, a major one being that it terminates in a superfamily suffix) are a group of about 6,000-7,000 species which are mainly ectoparasitic on fishes, especially on the gills and skin, and occasionally other aquatic organisms, such as amphibians. A small number of species also occur as endoparasites. The majority of monogenean species (Fig. 2) are highly host-specific, usually being restricted to a single host species with which they have co-evolved. A direct life-cycle using a ciliated larva for transmission is usual. However, gyrodactylids are unusual in that they are viviparous, and each worm gives birth to a fully-developed young worm which may have a third generation developing within it (polyembryony); thus their population size can increase very rapidly. In freshwater, the monogenean fauna is dominated by two huge genera, *Dactylogyrus* and *Gyrodactylus*, with 900+ and 400+ nominal species, respectively (Gibson et al. 1996; Harris et al. 2004). It is quite common to find more than one species of the same genus on the same host. Generally, monogeneans do little harm to their hosts, but some species, especially some gyrodactylids, can cause mass mortalities in fish-farms and/or situations where the parasite is introduced into an immunologically naive host population.

Monogeneans are generally distinguished by features of their posterior attachment organ (haptor), which is normally armed with attachment clamps and/or anchors and hooks, and in some cases the structure of the sclerotised hardparts of the male and female reproductive systems is also important. Such differences between congeneric species can be very subtle. Classifications vary, but those forms where the haptor is typically armed with clamps (or suckers) and minute (vestigial) hooks are referred to the subclass Polyopisthocotylea (or Oligonchoinea + Polystomatoinea), and those armed with hooks only (some large) belong to the subclass Monopisthocotylea (or Polyonchoinea). Most polyopisthocotyleans live on the gills and feed on blood, whereas most monopisthocotyleans live on the skin or gills and tend to feed on skin and/or mucus. Recent molecular work has suggested that these two groups are independent and that the Monogenea may not be monophyletic (Perkins et al. 2010). At lower levels, morphologically-based classifications, such as that of Boeger and Kritsky (1993), do not always agree with those based on molecular data (e.g. Šimková et al. 2006; Perkins et al. 2010), but some on-line data-bases (e.g. MonoDB (http://www.monodb.org/) have the potential and adaptability to develop a more integrated and useful system.

The **Trematoda** is a large class of 15,000-20,000 species which utilise all of the major vertebrate groups as hosts. Most trematodes (flukes) are endoparasitic as adults and live in the alimentary canal, but the group is extremely adaptable in terms of site, with different species occurring in most major body cavities and organs, and a very small number being ectoparasitic. One distinctive feature of virtually all trematodes is the involvement of molluscs in their life-history. There are two subclasses, the Aspidogastrea and the Digenea.

The **Aspidogastrea** is a small, disparate group of fewer than 100 species, whose members occur as gut parasites of molluscs, fishes and turtles. Those in molluscs have a direct life-cycle, whereas those with vertebrate hosts, where the complete life-cycle is known, use molluscs as primary hosts, with transmission by ingestion. They generally have a relatively low level of host-specificity. There are four families, all of which possess either a large, subdivided ventral disc or a row of suckers; only one family, the Aspidogastridae, occurs in freshwater, whereas the other three families are marine. A recent key to the genera can be found in Rohde (2002).

**Figure 3.**
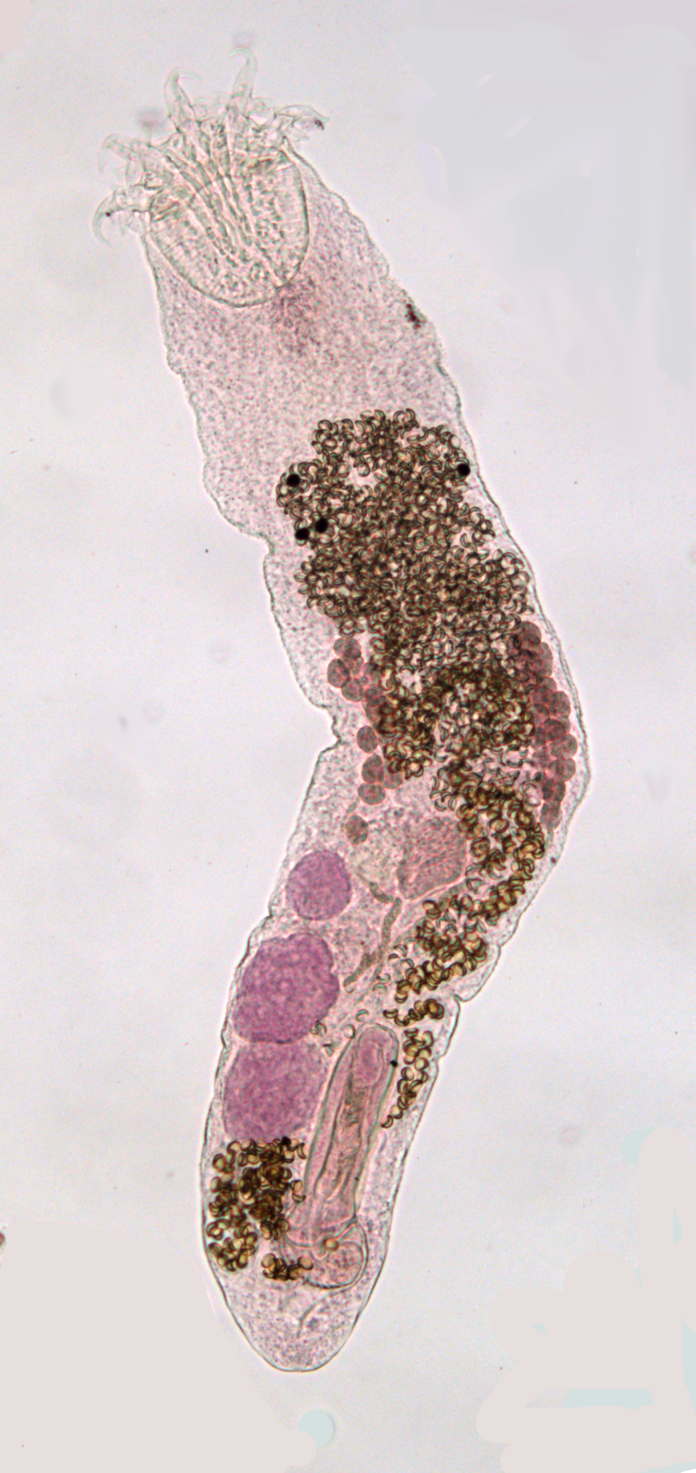
Bucephalid digenean.

The **Digenea** is an enormous group of more than 2,500 nominal genera (Gibson 2002). Virtually all use molluscs as primary hosts and the majority occur as sexual adults in vertebrates (all groups), but they are especially prevalent in fishes. Digeneans (Fig. 3) are characterised by having multiple generations (usually three) within their life-cycle – two asexual generations mature in the mollusc host and one sexual generation within a vertebrate host. The first generation, termed the mother-sporocyst, is derived from a ciliated larva, the miracidium, which develops within the egg and infects the mollusc host. The mother-sporocyst produces a second parthenogenetic generation internally, termed a daughter-sporocyst or redia, depending on its morphology. This second generation normally produces a large number of tailed, larval forms of the third, sexual, generation, called cercariae, which are free-living. Transmission to the next host is usually by cercarial penetration or by the ingestion of cercariae. In most cases the cercaria encysts within the tissues of a second (or intermediate) host, which may be an invertebrate or a vertebrate, as a resting stage, the metacercaria. This stage remains within this host until it is eaten by the final host, normally a vertebrate, within which the sexually mature, egg-laying adult develops.

Digeneans are thought to generally exhibit a high level of host-specificity to the mollusc host, a low level to any intermediate host and a variable level to the final host. The form of the life-cycle can be extremely plastic in the different groups; for example, in some the cercaria can encyst on vegetation and the herbivorous final host acquires the parasite in its diet, and, in others, the life-cycle is telescoped via the parasite maturing in a host that at one time during its evolution represented an intermediate host, or is extended by the addition of another vertebrate host via the ingestion of a host that was once the final host. With regard to their morphology, digeneans are even more diverse. Although the standard pattern is for a species to have a sucker at the anterior end and another on the ventral surface, some groups have one sucker and others none at all. Some have a body form which is totally unrecognisable as a digenean to a non-specialist. Other somewhat rare variations in structure are found in groups which have an intestine with an anus or ani, and others have no gut at all. There are rare dioecious forms, forms with the entire life-cycle in one and the same host and forms which live on the gills, in the vascular system or under fish scales. Classifications vary, but recent opinion indicates the presence of only two or three orders. Although there are recent molecular phylogenies (e.g. Olson et al. 2003), the most useful identification aids to the generic level are the three volumes of the ‘Keys to the Trematoda‘ (Gibson et al. 2002; Jones et al. 2005; Bray et al. 2008).

**Figure 4.**
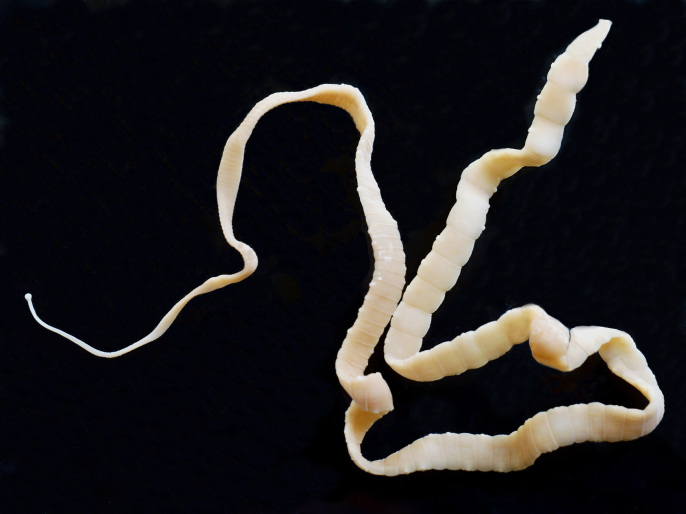
Taeniid cestode.

The **Cestoda** (tapeworms) is a relatively large (c. 8,000 species) and diverse group of parasites, the majority of which are found in the intestine of vertebrates (all groups). Like the acanthocephalans, they lack an alimentary canal and absorb their nutrients through their surface layer (tegument), which bears a dense covering of armed, villus-like structures (microtriches) that greatly increase its surface area and represent the main site for nutrient absorption. Tapeworms (Fig. 4) are also unusual in that the majority are long, tape-like and segmented, with one, or occasionally two, complete sets of reproductive organs in each segment. New segments (proglottids) are formed in the neck region behind the head (scolex); these develop and mature as they pass down the body (strobila) and old, ‘gravid’ segments containing eggs are lost terminally. A small number of basal forms that parasitise fishes (occasionally invertebrates) lack segmentation and possess only one or multiple sets of reproductive organs. Tapeworms vary in size from just a few millimetres to many metres in length. Since most adult tapeworms absorb nutrients though their tegument, they tend to do little physical damage to their host, except perhaps at the point of attachment, but they do extract valuable resources from the intestine and can cause bowel obstruction in the case of heavy infections. These worms do not roam freely in the intestine but attach to the wall of the intestine.

Cestodes generally have a life-cycle involving one or two intermediate hosts. Since adult cestodes are intestinal parasites of vertebrates, the eggs or gravid segments containing eggs pass out with the faeces. In those groups prevalent in terrestrial vertebrates, if the eggs are eaten by a suitable intermediate host, which may be a terrestrial invertebrate (commonly an arthropod) or a vertebrate, they hatch to release a hexacanth (six-hooked) larva, called an onchosphere. In the case of those groups more prevalent in fishes or other aquatic vertebrates, the eggs hatch in water to release a ciliated, motile hexacanth, called a coracidium, which is eaten by an aquatic arthropod intermediate host, such as a copepod. In the intermediate host, the hexacanth usually penetrates the gut wall and develops in the body-cavity into a procercoid. It then develops further, either in the same host or, in cases where the first host is eaten, in a second intermediate host, into a resting, normally encysted, stage, which takes on a variety of names, depending upon its form, e.g. cysticercus, cysticercoids or plerocercoid (Chervy 2002). The definitive host acquires the parasite when it feeds on the intermediate host harbouring the encysted stage.

There are marked differences in the form of the attachment organ on the scolex, which form the main criteria for distinguishing the numerous (*c.* 15) orders of the group. Other important characters include the shape of the segments and the arrangement and form of the reproductive system(s) within the segments, e.g. the position of the genital pore, the nature of the vitellarium, the size of the cirrus-sac, the shape of the ovary and the nature of the uterus. Some of these features are also used to distinguish genera. At the specific level, the number and morphometrics of the hooks, which commonly form the armature of the scolex, are useful. The functional classification of the group is still based on morphology, but, although the basic arrangement is rather stable, molecular data indicate that some changes are needed. The ‘Keys to the Cestode Parasites of Vertebrates’ (Khalil et al. 1994) provides keys down the generic level, and a molecular classification was given by Olson et al. (2001), with updates by Waeschenbach et al. (2007) and Waeschenbach et al. (2012). Recent work (Kuchta et al. 2008a; Kuchta et al. 2008b) has shown that one of the major and important orders (the Pseudophyllidea) is not monophyletic – this has been replaced by two new orders (the Bothriocephalidea and the Diphyllobothriidea). In addition, a large order of shark parasites, the Tetraphyllidea, has been shown by molecular studies to be paraphyletic. The dismemberment of this taxon is now underway.

**Figure 5.**
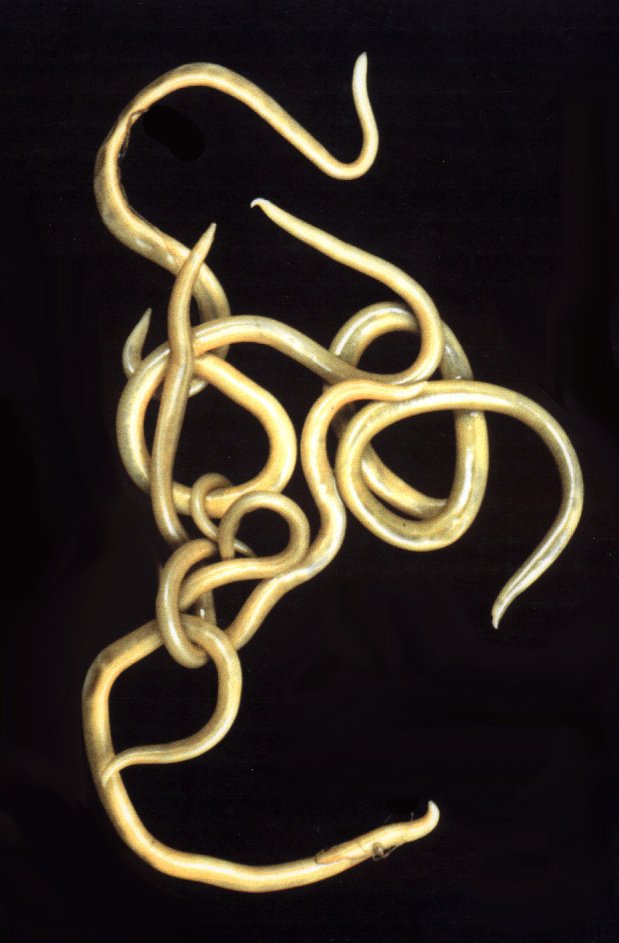
Anisakid nematode.

The phylum **NEMATODA** is probably the most abundant and widespread animal group, often occurring in huge numbers in environments ranging from hot springs to polar regions. In addition to free-living marine and freshwater forms, there are free-living forms in the soil and parasitic forms in both animals and plants. At least 30,000 species are known, but this is estimated to be only a very small fraction of those that exist. Nematodes (Fig. 5) are symmetrically bilateral, unsegmented, normally dioecious worms which are usually filiform in shape. Their main features include a body-cavity with a high hydrostatic pressure, a straight digestive tract with an anteriorly terminal mouth and posteriorly subterminal anus, no circulatory system, a simple excretory system and a body wall consisting of an outer layer of cuticle and an inner layer of longitudinal muscles. Those parasitic in animals occur in virtually all invertebrate and vertebrate groups. All nematodes have five life-history stages, four larval and one adult, which are separated by a moult of the cuticle. It is common for the first one or two moults to occur within the egg. The free-living and plant-parasitic members of the group are dealt with elsewhere.

The phylum is divided into two classes, the Adenophorea and the Secernentea, both of which have evolved parasitic members, although the majority of animal parasites belong to the latter group. Major differences between the groups reflect the presence and absence of small sensory structures (phasmids) on the tail and the nature of the excretory system. There is also a fundamental biological difference in the parasitic members, since in adenophoreans the first-stage larva is infective to the definitive (final) host, whereas in the Secernentea it is the third-stage larva.

The life-cycles of parasitic forms may be direct or indirect. Direct life-cycles may involve the ingestion of eggs or larvae with food or, in some cases, the direct penetration of larvae through the skin. Indirect life-cycles usually utilise invertebrate intermediate hosts, but sometimes vertebrates (or larger invertebrates) may act as intermediate or paratenic hosts. Such larvae usually occur, often encysted, in the tissues of intermediate hosts. The majority of nematodes parasitic in vertebrates occur in the alimentary canal; those in other parts of the body often require the migration of larvae through the body to reach these sites. Some groups with a direct life-cycle also have a larval migration from the gut and into the tissues and back to the gut; this represents the vestige of an indirect life-cycle from its evolutionary past. Whichever mode of transmission is utilised, the chance of an egg or larva developing into an adult worm is very small, but this may be compensated for by a huge output of eggs, which in some cases reaches as high as 200,000 per day from a single female worm.

Pathogenicity in the definitive host varies considerably, usually being dependent upon the size of the infection. Those, such as hookworms, which are heavily armed with teeth or other sclerotised mouthparts and browse upon the gut wall, can cause considerable damage. Similarly, forms which migrate around the body, both as adults in the tissues and as larvae (the latter termed a larva migrans), can cause serious problems, especially if they reach sensitive regions such as the brain, liver or eyes.

Features used for identification vary from group to group, but at higher taxonomic levels, the nature of the oesophagus, the form of the head (presence and number of lips, teeth, etc.) and the form of the male tail are usually important. At the specific level, details of the male tail, such as the arrangement of caudal papillae (sensory structures used during copulation) and the length and shape of the spicule or spicules (sclerotised copulatory aids) are important. In most cases, males carry more taxonomically useful information than females, such that the latter are often unidentifiable at the specific level. The most used classification of the nematodes based on morphology is that of ‘Keys to the Nematode Parasites of Vertebrates’, published as a series of 10 booklets between 1974 and 1983. This has recently been re-issued as a single volume (Anderson et al. 2009) and updated by Gibbons (2010). A similar key to the nematode parasites of invertebrates was produced by Poinar (1977). These volumes include keys to the generic level. There are also molecular versions of the classifications of the entire phylum (e.g. Meldal et al. 2007) and of various subgroups (e.g. Nadler and Hudspeth 2000).

## Project description

### Title

This BDJ data paper includes the taxonomic indexing efforts in the Fauna Europaea on European helminths covering the first two versions of Fauna Europaea worked on between 2000 and 2013 (up to version 2.6).

### Personnel

The taxonomic framework of Fauna Europaea includes partner institute, providing taxonomic expertise and information, and expert networks taking care of data collation.

Every taxonomic group is covered by at least one Group Coordinator responsible for the supervision and integrated input of taxonomic and distributional data for a particular group. For helminths, the responsible Group Coordinator is David Gibson (versions 1 & 2).

The Fauna Europaea checklist would not have reached its current level of completion without the input from several groups of specialists. The formal responsibility of collating and delivering the data for the relevant families rested with a number of Taxonomic Specialists (see Table 1). Associate Specialists deserve credit for their important contributions at various levels, including for particular geographical regions or across taxonomic groups (see Table 2).

An overview of the expert network for helminths can be found here: http://www.faunaeur.org/experts.php?id=51.

Data management tasks are taken care of by the Fauna Europaea project bureau. During the project phase (until 2004) a network of principal partners took care of diverse management tasks: the Zoological Museum Amsterdam (general management & system development), the Zoological Museum of Copenhagen (data collation), the National Museum of Natural History in Paris (data validation) and the Museum and Institute of Zoology in Warsaw (NAS extension). Since the formal project ended (2004-2013), all tasks have been undertaken by the Zoological Museum Amsterdam.

### Study area description

The area study covers the European mainland (Western Palaearctic), including the Macaronesian islands, and excluding the Caucasus, Turkey, the Arabian Peninsula and Northern Africa.

### Design description

Standards. Group coordinators and taxonomic specialists have had to deliver the (sub)species names according to strict standards. The names provided by the Fauna Europaea (FaEu) are scientific names. The taxonomic scope includes issues such as: (1) the definition of criteria used to identify accepted species-group taxa; (2) the hierarchy (classification scheme) for the accommodation of all accepted species and (3) relevant synonyms; and (4) the correct nomenclature. The Fauna Europaea 'Guidelines for Group Coordinators and Taxonomic Specialists' includes the standards, protocols, scope and limits that provide instructions for all of the more then 400 specialists contributing to the project.

Data management. The data records could either be entered offline into a preformatted MS-Excel worksheet or directly into the Fauna Europaea transaction database using an online browser interface. The data servers are hosted at the University of Amsterdam.

Data set. The Fauna Europaea basic data set consists of: accepted (sub)species names (including authorship), synonym names (including authorship), a taxonomic hierarchy/classification, misapplied names (including misspellings and alternative taxonomic views), homonym annotations, expert details, European distribution (at the country level), Global distribution (only for European species), taxonomic reference (optional), and occurrence reference (optional).

### Funding

Fauna Europaea was funded by the European Commission under various framework programs (see above).

## Sampling methods

### Study extent

See spatial coverage and geographical coverage descriptions.

### Sampling description

Fauna Europaea data have been assembled by principal taxonomic experts, based on their individual expertise, including literature sources, collection research and field observations. In total no less than 476 experts contributed taxonomic and/or faunistic information for the Fauna Europaea. The vast majority of the experts are from Europe (including EU non-member states). As a unique feature, Fauna Europaea funds were set aside for rewarding/compensating for the work of taxonomic specialists and group coordinators.

**Figure 6.**
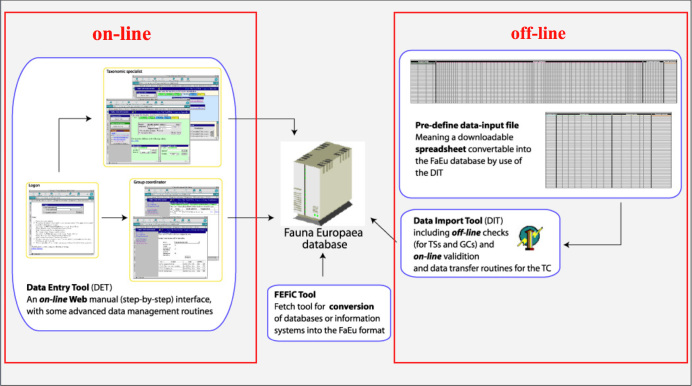
Fauna Europaea on-line (browser interfaces) and off-line (spreadsheets) data entry tools.

To facilitate data transfer and data import, sophisticated on-line (web interfaces) and off-line (spreadsheets) data-entry routines were built and integrated within an underlying central Fauna Europaea transaction database (see Fig. 6). This includes advanced batch data import routines and utilities to display and monitor the data processing within the system. In retrospect, it seems that the off-line submission of data was probably the best for bulk import during the project phase, whereas the on-line tool was preferred to enter modifications in later versions. This system has worked well but may be replaced in 2013.

A first release of the Fauna Europaea index via the web-portal has been presented at 27 ^th^ of September 2004, the most recent release (version 2.6.2) was launched at 29 August 2013. An overview of Fauna Europaea releases can be found here: http://www.faunaeur.org/about_fauna_versions.php.

### Quality control

Fauna Europaea data are unique in the sense that they are fully expert based. Selecting leading experts for all groups assured the systematic reliability and consistency of the Fauna Europaea data.

Furthermore, all Fauna Europaea data sets are intensively reviewed at regional and thematic validation meetings, at review sessions at taxonomic symposia (for some groups), by Fauna Europaea Focal Points (during the FaEu-NAS and PESI projects) and by various end-users sending annotations using a web form at the web-portal. Additional validation of gaps and correct spelling was effected at the validation office in Paris.

In conclusion, we expect to get taxonomic data for 99.3% of the known European fauna. The faunistic coverage is not quite as good, but nevertheless represents 90-95% of the total fauna. Recognised gaps for the helminths include: areas where the geographical divisions of the Host-Parasite Database of the Natural History Museum, London (the main source of the data), as outlined below, are not concordant with those of the Fauna Europaea. It is also likely that, although an update to include new taxa was carried out in 2006, the inclusion of recent geographical data may be limited.

Checks on the technical and logical correctness of the data have been implemented in the data entry tools, including around 50 business rules (http://dev.e-taxonomy.eu/trac/wiki/IntegrityRulesEditPESI). This validation tool proved to be of huge value for both the experts and project management, and it contributed significantly to the preparation of a remarkably clean and consistent data set. This thorough reviewing makes Fauna Europaea the most scrutinised data sets in its domain.

### Step description

By evaluating the team structure and life-cycle procedures (data-entry, validation, updating, etc.), clear definitions of the roles of users and user-groups, depending on the taxonomic framework, were established, including ownership and read and writes privileges, and their changes during the project life-cycle. In addition, guidelines on common data exchange formats and codes have been issued.

## Geographic coverage

### Description

**Figure 7.**
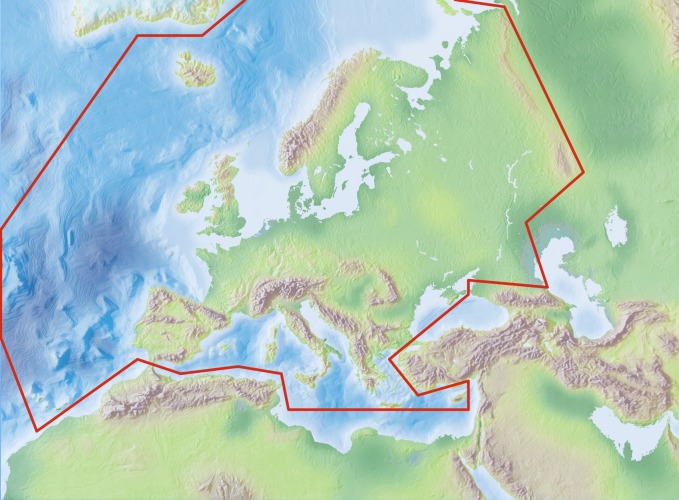
Fauna Europaea geographical coverage ('minimal Europe').

Species and subspecies distributions in the Fauna Europaea are registered at least at the country level, i.e. for political countries. For this purpose, the FaEu geographical system basically follows the TDWG standards. The covered area includes the European mainland (Western Palaearctic), plus the Macaronesian islands (excl. Cape Verde Islands), Cyprus, Franz Josef Land and Novaya Zemlya. Western Kazakhstan and the Caucasus are excluded (see Fig. 7).

The focus is on species (or subspecies) of European multicellular animals of terrestrial and freshwater environments. Species in brackish waters, occupying the marine/freshwater or marine/terrestrial transition zones, are generally excluded.

A large proportion of the helminth records for this compilation was acquired from the Host-Parasite Database maintained by the Parasitic Worms Group at the Natural History Museum (NHM) in London. These data were supplemented by searches of primary and secondary literature sources and by information supplied by specialists who checked sections of the files.

There are some geographical discrepancies between the data acquired from the NHM Database and that required for the FaEu files. These are:

The Czech and Slovak Republics are included as Czechoslovakia.

The recent subdivision of Yugoslavia is not implemented.

The Irish Republic and Northern Ireland are combined.

The Ukraine and Moldova are combined.

Estonia and Latvia are combined.

European and Asian Turkey are not distinguished.

Russia is not subdivided.

In some cases these data have been adapted, but records from these areas should be treated with caution.

### Coordinates

Mediterranean (N 35°) and Arctic Islands (N 82°) Latitude; Atlantic Ocean (Mid-Atlantic Ridge) (W 30°) and Urals (E 60°) Longitude.

## Taxonomic coverage

### Description

The Fauna Europaea database contains the scientific names of all living European land and freshwater animal species, including numerous infra-groups and synonyms. More details of the conceptual background of Fauna Europaea and standards followed are described in the project description papers.

This data paper covers the Helminth (animal parasitic) content of Fauna Europaea, including 214 families, 3986 species, 32 subspecies and 435 (sub)species synonyms (see FaEu Helminths stats for a species per family chart.)

### Taxa included

**Table:** 

Rank	Scientific Name	Common Name
kingdom	Animalia	
subkingdom	Eumetazoa	
phylum	Acanthocephala	
phylum	Platyhelminthes	
phylum	Nematoda	
subphylum	Neodermata	
class	Archiacanthocephala	
class	Cestoda	
class	Eoacanthocephala	
class	Monogenea	
class	Palaeacanthocephala	
class	Trematoda	
subclass	Aspidogastrea	
subclass	Digenea	
subclass	Monopisthocotylea	
subclass	Polyopisthocotylea	
superorder	Oligonchoinea	
superorder	Polyonchoinea	
superorder	Polystomatoinea	
order	Amphilinidea	
kingdom	Apororhynchida	
order	Ascaridida	
order	Aspidogastrida	
order	Capsalidea	
order	Caryophyllidea	
order	Cyclophyllidea	
order	Dactylogyridea	
order	Diclybothriidea	
order	Echinorhynchida	
order	Echinostomida	
order	Gigantorhynchida	
order	Gyracanthocephala	
order	Gyrodactylidea	
order	Mazocreaidea	
order	Moniliformida	
order	Neoechinorhynchida	
order	Oligacanthorhynchida	
order	Oxyurida	
order	Plagiorchiida	
order	Polymorphida	
order	Proteocephalidea	
order	Pseudophyllidea [now Bothriocephalidea and Diphyllobothriidea]	
order	Spathebothriidea	
order	Strigeida	
order	Strongylida	
order	Tetrabothriidea	
suborder	Aphelenchina	
suborder	Dactylogyrinea	
suborder	Discocotylinea	
suborder	Hexatylina	
suborder	Mazocraeinea	
suborder	Microcotylinea	
suborder	Oxyurina	
suborder	Tetraonchinea	
superfamily	Allocreadioidea	
superfamily	Ancylostomatoidea	
superfamily	Aphelenchoidea	
superfamily	Ascaridoidea	
superfamily	Clinostomoidea	
superfamily	Cosmoceroidea	
superfamily	Cyclocoeloidea	
superfamily	Diaphanocephaloidea	
superfamily	Diplogasteroidea	
superfamily	Diplogastroidea	
superfamily	Diplostomoidea	
superfamily	Drilonematoidea	
superfamily	Echinostomatoidea	
superfamily	Gymnophalloidea	
superfamily	Hemiuroidea	
superfamily	Heterakoidea	
superfamily	Iotonchioidea	
superfamily	Lepocreadioidea	
superfamily	Mermithoidea	
superfamily	Metastrongyloidea	
superfamily	Microphalloidea	
superfamily	Notocotyloidea	
superfamily	Opisthorchioidea	
superfamily	Oxyuroidea	
superfamily	Paramphistomoidea	
superfamily	Plagiorchioidea	
superfamily	Rhabditoidea	
superfamily	Schistosomoidea	
superfamily	Seuratoidea	
superfamily	Sphaerularioidea	
superfamily	Strongyloidea	
superfamily	Subuluroidea	
superfamily	Tetradonematoidea	
superfamily	Thelastomatoidea	
superfamily	Trichostrongyloidea	
superfamily	Troglotrematoidea	
superfamily	Zoogonoidea	
family	Acanthocolpidae	
family	Acanthostomidae [now syn. of Cryptogonimidae]	
family	Acoleidae	
family	Acrobothriidae	
family	Acuariidae	
family	Agfidae	
family	Alirhabditidae	
family	Allantonematidae	
family	Allocreadiidae	
family	Amabiliidae	
family	Amidostomidae	
family	Amphilinidae	
family	Ancylostomatidae	
family	Ancyrocephalidae	
family	Angiostomatidae	
family	Angiostrongylidae	
family	Anguillicolidae	
family	Anisakidae	
family	Anoplocephalidae	
family	Apororhynchidae	
family	Aproctidae	
family	Arhythmacanthidae	
family	Ascarididae	
family	Ascaridiidae	
family	Aspidogastridae	
family	Atractidae	
family	Auridistomidae	
family	Azygiidae	
family	Bolbocephalodidae [treated as syn. of Strigeidae, but now recognised]	
family	Bothriocephalidae	
family	Brachycoeliidae	
family	Brachylaimidae	
family	Bucephalidae	
family	Bunocotylidae	
family	Camallanidae	
family	Capillariidae	
family	Capsalidae	
family	Carabonematidae	
family	Caryophyllaeidae	
family	Catenotaeniidae	
family	Cathaemasiidae	
family	Centrorhynchidae	
family	Cephalochlamydidae	
family	Cephalogonimidae	
family	Chabertiidae	
family	Cladorchiidae	
family	Clinostomidae	
family	Collyriclidae	
family	Cosmocercidae	
family	Crenosomatidae	
family	Cryptogonimidae	
family	Cucullanidae	
family	Cyathocotylidae	
family	Cyclocoelidae	
family	Cystidicolidae	
family	Cystoopsidae	
family	Dactylogyridae	
family	Daniconematidae	
family	Davaineidae	
family	Derogenidae	
family	Deropristidae [treated as syn. of Acanthocolpidae, but now recognised]	
family	Desmidocercidae	
family	Diaphanocephalidae	
family	Diclybothriidae	
family	Dicrocoeliidae	
family	Dictyocaulidae	
family	Dilepididae	
family	Dioctophymatidae	
family	Dioecocestidae	
family	Diphyllobothriidae	
family	Diplectanidae	
family	Diplodiscidae	
family	Diplostomidae	
family	Diplotriaenidae	
family	Diplozoidae	
family	Dipylidiidae	
family	Discocotylidae	
family	Dracunculidae	
family	Drilonematidae	
family	Echinorhynchidae	
family	Echinostomatidae	
family	Ektaphelenchidae	
family	Entaphelenchidae	
family	Eucotylidae	
family	Eumegacetidae	
family	Fasciolidae	
family	Faustulidae	
family	Filariidae	
family	Filaroididae	
family	Gastrodiscidae	
family	Gastrothylacidae	
family	Gigantorhynchidae	
family	Gnathostomatidae	
family	Gongylonematidae	
family	Gorgoderidae	
family	Gymnophallidae	
family	Gyrodactylidae	
family	Habronematidae	
family	Haplometridae [syn. of Plagiorchiidae]	
family	Haploporidae	
family	Haplosplanchnidae	
family	Hartertiidae	
family	Hedruridae	
family	Heligmonellidae	
family	Heligmosomidae	
family	Hemiuridae	
family	Heterakidae	
family	Heterophyidae	
family	Heterorhabditidae	
family	Heteroxynematidae	
family	Hymenolepididae	
family	Iagotrematidae	
family	Illiosentidae	
family	Iotonchiidae	
family	Kathlaniidae	
family	Kiwinematidae	
family	Lecithasteridae	
family	Lecithodendriidae	
family	Leucochloridiidae	
family	Leucochloridiomorphidae	
family	Lytocestidae	
family	Macroderidae	
family	Mazocraeidae	
family	Mermithidae	
family	Mesocestoididae	
family	Mesotretidae	
family	Metadilepididae	
family	Metastrongylidae	
family	Microcotylidae	
family	Microphallidae	
family	Molineidae	
family	Moniliformidae	
family	Monorchiidae	
family	Muspiceidae	
family	Nanophyetidae	
family	Nematotaeniidae	
family	Neoechinorhynchidae	
family	Notocotylidae	
family	Octomacridae	
family	Oligacanthorhynchidae	
family	Omphalometridae	
family	Onchocercidae	
family	Opecoelidae	
family	Opisthorchiidae	
family	Orchipedidae	
family	Ornithostrongylidae	
family	Oxyuridae	
family	Pachypsolidae	
family	Panopistidae	
family	Paramphistomidae	
family	Parasitaphelenchidae	
family	Parasitylenchidae	
family	Paruterinidae	
family	Paurodontidae	
family	Pharyngodonidae	
family	Philometridae	
family	Philophthalmidae	
family	Physalopteridae	
family	Plagiorchiidae	
family	Plagiorchiidae	
family	Plagiorhynchidae	
family	Pneumospiruridae	
family	Polymorphidae	
family	Polystomatidae	
family	Pomphorhynchidae	
family	Progynotaeniidae	
family	Pronocephalidae	
family	Prosthogonimidae	
family	Proteocephalidae	
family	Protostrongylidae	
family	Pseudaliidae	
family	Pseudonymidae	
family	Psilostomidae	
family	Quadrigyridae	
family	Quimperiidae	
family	Renicolidae	
family	Rhabdiasidae	
family	Rhabdochonidae	
family	Rhadinorhynchidae	
family	Rictulariidae	
family	Robertdollfusidae	
family	Sanguinicolidae	
family	Schistosomatidae	
family	Seuratidae	
family	Skrjabillanidae	
family	Skrjabingylidae	
family	Soboliphymatidae	
family	Sphaerulariidae	
family	Spirocercidae	
family	Spirorchiidae	
family	Spiruridae	
family	Steinernematidae	
family	Stomylotrematidae	
family	Strigeidae	
family	Strigeidae	
family	Strongylacanthidae	
family	Strongylidae	
family	Strongyloididae	
family	Subuluridae	
family	Syngamidae	
family	Syrphonematidae	
family	Taeniidae	
family	Telorchiidae	
family	Tenuisentidae	
family	Tetrabothriidae	
family	Tetrameridae	
family	Tetraonchidae	
family	Thapariellidae	
family	Thelastomatidae	
family	Thelaziidae	
family	Travassosinematidae	
family	Triaenophoridae	
family	Trichinellidae	
family	Trichosomoididae	
family	Trichostrongylidae	
family	Trichuridae	
family	Troglotrematidae	
family	Typhlocoelidae	
family	Zoogonidae	

## Temporal coverage

**Living time period:** Currently living animals in stable populations, largely excluding (1) rare/irregular immigrants, intruder or invader species, (2) accidental or deliberate releases of exotic (pet) species, (3) domesticated animals, (4) foreign species imported and released for bio-control or (5) foreign species largely confined to hothouses.

## Usage rights

### Use license

Other

### IP rights notes

The Fauna Europaea license for use is CC BY.

For more IPR details see: http://www.faunaeur.org/copyright.php.

## Data resources

### Data package title

Fauna Europaea - Helminths (Animal Parasitic)

### Resource link

http://www.faunaeur.org/Data_papers/FaEu_Helminths_2.6.2.zip

### Alternative identifiers

http://www.faunaeur.org/full_results.php?id=16175

### Number of data sets

2

### Data set 1.

#### Data set name

Fauna Europaea - Helminths (Animal Parasitic) version 2.6.2 - species

#### Data format

CSV

#### Number of columns

24

#### Character set

UTF-8

#### Download URL

http://www.faunaeur.org/Data_papers/FaEu_Helminths_2.6.2.zip

#### Description

**Data set 1.** 

Column label	Column description
datasetName	The name identifying the data set from which the record was derived (http://rs.tdwg.org/dwc/terms/datasetName).
version	Release version of data set.
versionIssued	Issue data of data set version.
rights	Information about rights held in and over the resource (http://purl.org/dc/terms/rights).
rightsHolder	A person or organization owning or managing rights over the resource (http://purl.org/dc/terms/rightsHolder).
accessRights	Information about who can access the resource or an indication of its security status (http://purl.org/dc/terms/accessRights).
taxonID	An identifier for the set of taxon information (http://rs.tdwg.org/dwc/terms/taxonID)
parentNameUsageID	An identifier for the name usage of the direct parent taxon (in a classification) of the most specific element of the scientificName (http://rs.tdwg.org/dwc/terms/parentNameUsageID).
scientificName	The full scientific name, with authorship and date information if known (http://rs.tdwg.org/dwc/terms/scientificName).
acceptedNameUsage	The full name, with authorship and date information if known, of the currently valid (zoological) taxon (http://rs.tdwg.org/dwc/terms/acceptedNameUsage).
originalNameUsage	The original combination (genus and species group names), as firstly established under the rules of the associated nomenclaturalCode (http://rs.tdwg.org/dwc/terms/originalNameUsage).
family	The full scientific name of the family in which the taxon is classified (http://rs.tdwg.org/dwc/terms/family).
familyNameId	An identifier for the family name.
genus	The full scientific name of the genus in which the taxon is classified (http://rs.tdwg.org/dwc/terms/genus).
subgenus	The full scientific name of the subgenus in which the taxon is classified. Values include the genus to avoid homonym confusion (http://rs.tdwg.org/dwc/terms/subgenus).
specificEpithet	The name of the first or species epithet of the scientificName (http://rs.tdwg.org/dwc/terms/specificEpithet).
infraspecificEpithet	The name of the lowest or terminal infraspecific epithet of the scientificName, excluding any rank designation (http://rs.tdwg.org/dwc/terms/infraspecificEpithet).
taxonRank	The taxonomic rank of the most specific name in the scientificName (http://rs.tdwg.org/dwc/terms/infraspecificEpithet).
scientificNameAuthorship	The authorship information for the scientificName formatted according to the conventions of the applicable nomenclaturalCode (http://rs.tdwg.org/dwc/terms/scientificNameAuthorship).
authorName	Author name information
namePublishedInYear	The four-digit year in which the scientificName was published (http://rs.tdwg.org/dwc/terms/namePublishedInYear).
Brackets	Annotation if authorship should be put between parentheses.
nomenclaturalCode	The nomenclatural code under which the scientificName is constructed (http://rs.tdwg.org/dwc/terms/nomenclaturalCode).
taxonomicStatus	The status of the use of the scientificName as a label for a taxon (http://rs.tdwg.org/dwc/terms/taxonomicStatus).

### Data set 2.

#### Data set name

Fauna Europaea - Helminths (Animal Parasitic) version 2.6.2 - hierarchy

#### Data format

CSV

#### Number of columns

11

#### Character set

UTF-8

#### Download URL

http://www.faunaeur.org/Data_papers/FaEu_Helminths_2.6.2.zip

#### Description

**Data set 2.** 

Column label	Column description
datasetName	The name identifying the data set from which the record was derived (http://rs.tdwg.org/dwc/terms/datasetName).
version	Release version of data set.
versionIssued	Issue data of data set version.
rights	Information about rights held in and over the resource (http://purl.org/dc/terms/rights).
rightsHolder	A person or organization owning or managing rights over the resource (http://purl.org/dc/terms/rightsHolder).
accessRights	Information about who can access the resource or an indication of its security status (http://purl.org/dc/terms/accessRights).
taxonName	The full scientific name of the higher-level taxon
scientificNameAuthorship	The authorship information for the scientificName formatted according to the conventions of the applicable nomenclaturalCode (http://rs.tdwg.org/dwc/terms/scientificNameAuthorship).
taxonRank	The taxonomic rank of the most specific name in the scientificName (http://rs.tdwg.org/dwc/terms/infraspecificEpithet).
taxonID	An identifier for the set of taxon information (http://rs.tdwg.org/dwc/terms/taxonID)
parentNameUsageID	An identifier for the name usage of the direct parent taxon (in a classification) of the most specific element of the scientificName (http://rs.tdwg.org/dwc/terms/parentNameUsageID).

## Figures and Tables

**Table 1.** Specialists responsible for each helminth family.

FAMILY	NUMBER OF SPECIES	SPECIALIST(S)
Acanthocolpidae	3	David Gibson
Acanthostomidae	2	David Gibson
Acoleidae	4	Rodney Bray
Acrobothriidae	5	Rodney Bray
Acuariidae	96	David Gibson
Agfidae	2	David J. Hunt
Alirhabditidae	1	David Gibson
Allantonematidae	78	David J. Hunt
Allocreadiidae	15	David Gibson
Amabiliidae	13	Rodney Bray
Amidostomidae	12	David Gibson
Amphilinidae	1	Rodney Bray
Ancylostomatidae	12	David Gibson
Ancyrocephalidae	34	Rodney Bray
Angiostomatidae	5	David Gibson
Angiostrongylidae	15	David Gibson
Anguillicolidae	2	David Gibson
Anisakidae	30	David Gibson
Anoplocephalidae	49	Rodney Bray
Apororhynchidae	2	Rodney Bray
Aproctidae	11	David Gibson
Arhythmacanthidae	3	David Gibson
Ascarididae	34	David Gibson
Ascaridiidae	10	David Gibson
Aspidogastridae	2	David Gibson
Atractidae	4	David Gibson
Auridistomidae	2	David Gibson
Azygiidae	3	David Gibson
Bothriocephalidae	9	Rodney Bray
Brachycoeliidae	1	David Gibson
Brachylaimidae	28	David Gibson
Bucephalidae	5	David Gibson
Bunocotylidae	5	David Gibson
Camallanidae	5	David Gibson
Capillariidae	121	David Gibson
Capsalidae	1	Rodney Bray
Carabonematidae	1	David J. Hunt
Caryophyllaeidae	15	Rodney Bray
Catenotaeniidae	7	Rodney Bray
Cathaemasiidae	2	David Gibson
Centrorhynchidae	23	David Gibson
Cephalochlamydidae	1	Rodney Bray
Cephalogonimidae	2	David Gibson
Chabertiidae	10	David Gibson
Cladorchiidae	6	David Gibson
Clinostomidae	4	David Gibson
Collyriclidae	4	David Gibson
Cosmocercidae	15	David J. Hunt
Crenosomatidae	13	David Gibson
Cryptogonimidae	1	David Gibson
Cucullanidae	7	David Gibson
Cyathocotylidae	19	Rodney Bray
Cyclocoelidae	28	David Gibson
Cystidicolidae	12	David Gibson
Cystoopsidae	1	David Gibson
Dactylogyridae	125	Rodney Bray
Daniconematidae	1	David Gibson
Davaineidae	66	Rodney Bray
Derogenidae	4	David Gibson
Desmidocercidae	3	David Gibson
Diaphanocephalidae	3	David Gibson
Diclybothriidae	1	Rodney Bray
Dicrocoeliidae	88	David Gibson
Dictyocaulidae	8	David Gibson
Dilepididae	201	Rodney Bray
Dioctophymatidae	9	David Gibson
Dioecocestidae	4	Rodney Bray
Diphyllobothriidae	23	Rodney Bray
Diplectanidae	1	Rodney Bray
Diplodiscidae	1	David Gibson
Diplostomidae	67	David Gibson
Diplotriaenidae	22	Rodney Bray
Diplozoidae	20	Rodney Bray
Dipylidiidae	11	Rodney Bray
Discocotylidae	1	Rodney Bray
Dracunculidae	4	David Gibson
Drilonematidae	9	David J. Hunt
Echinorhynchidae	22	David Gibson
Echinostomatidae	153	David Gibson
Ektaphelenchidae	14	David J. Hunt
Entaphelenchidae	7	David J. Hunt
Eucotylidae	12	David Gibson
Eumegacetidae	10	David Gibson
Fasciolidae	5	David Gibson
Faustulidae	2	David Gibson
Filariidae	7	David Gibson
Filaroididae	7	David Gibson
Gastrodiscidae	1	David Gibson
Gastrothylacidae	1	David Gibson
Gigantorhynchidae	12	David Gibson
Gnathostomatidae	7	David Gibson
Gongylonematidae	12	David Gibson
Gorgoderidae	30	David Gibson
Gymnophallidae	19	David Gibson
Gyrodactylidae	117	Rodney Bray
Habronematidae	24	David Gibson
Haploporidae	5	David Gibson
Haplosplanchnidae	1	David Gibson
Hartertiidae	4	David Gibson
Hedruridae	1	David Gibson
Heligmonellidae	10	David Gibson
Heligmosomidae	26	David Gibson
Hemiuridae	9	David Gibson
Heterakidae	11	David Gibson
Heterophyidae	54	David Gibson
Heterorhabditidae	4	David J. Hunt
Heteroxynematidae	14	David Gibson
Hymenolepididae	343	Rodney Bray
Iagotrematidae	2	David Gibson
Illiosentidae	3	David Gibson
Iotonchiidae	9	David Gibson
Kathlaniidae	5	David Gibson
Kiwinematidae	2	David Gibson
Lecithasteridae	1	David Gibson
Lecithodendriidae	89	David Gibson
Leucochloridiidae	15	David Gibson
Leucochloridiomorphidae	2	David Gibson
Lytocestidae	9	Rodney Bray
Macroderidae	1	David Gibson
Mazocraeidae	1	Rodney Bray
Mermithidae	34	David J. Hunt
Mesocestoididae	12	Rodney Bray
Mesotretidae	1	David Gibson
Metadilepididae	3	Rodney Bray
Metastrongylidae	7	David Gibson
Microcotylidae	2	Rodney Bray
Microphallidae	54	David Gibson
Molineidae	41	David Gibson
Moniliformidae	3	David Gibson
Monorchiidae	13	David Gibson
Muspiceidae	2	David Gibson
Nanophyetidae	3	David Gibson
Nematotaeniidae	4	Rodney Bray
Neoechinorhynchidae	4	David Gibson
Notocotylidae	39	David Gibson
Octomacridae	1	Rodney Bray
Oligacanthorhynchidae	15	David Gibson
Omphalometridae	2	David Gibson
Onchocercidae	67	David Gibson
Opecoelidae	21	David Gibson
Opisthorchiidae	35	David Gibson
Orchipedidae	5	David Gibson
Ornithostrongylidae	3	David Gibson
Oxyuridae	28	David Gibson
Pachypsolidae	1	David Gibson
Panopistidae	5	David Gibson
Paramphistomidae	11	David Gibson
Parasitaphelenchidae	41	David J. Hunt
Parasitylenchidae	34	David J. Hunt
Paruterinidae	40	Rodney Bray
Paurodontidae	1	David J. Hunt
Pharyngodonidae	43	David Gibson
Philometridae	10	David Gibson
Philophthalmidae	24	David Gibson
Physalopteridae	23	David Gibson
Plagiorchiidae	91	David Gibson
Plagiorhynchidae	17	David Gibson
Pneumospiruridae	1	David Gibson
Polymorphidae	28	David Gibson
Polystomatidae	14	Rodney Bray
Pomphorhynchidae	5	David Gibson
Progynotaeniidae	8	Rodney Bray
Pronocephalidae	4	David Gibson
Prosthogonimidae	12	David Gibson
Proteocephalidae	24	Rodney Bray
Protostrongylidae	25	David Gibson
Pseudaliidae	2	David Gibson
Pseudonymidae	4	David J. Hunt
Psilostomidae	23	David Gibson
Quadrigyridae	1	David Gibson
Quimperiidae	2	David Gibson
Renicolidae	27	David Gibson
Rhabdiasidae	11	David Gibson
Rhabdochonidae	10	David Gibson
Rhadinorhynchidae	1	David Gibson
Rictulariidae	13	David Gibson
Robertdollfusidae	2	David Gibson
Sanguinicolidae	6	David Gibson
Schistosomatidae	19	David Gibson
Seuratidae	6	David Gibson
Skrjabillanidae	8	David Gibson
Skrjabingylidae	2	David Gibson
Soboliphymatidae	3	David Gibson
Sphaerulariidae	3	David J. Hunt
Spirocercidae	10	David Gibson
Spirorchiidae	1	David Gibson
Spiruridae	6	David Gibson
Steinernematidae	9	David J. Hunt
Stomylotrematidae	3	David Gibson
Strigeidae	45	David Gibson
Strongylacanthidae	2	David Gibson
Strongylidae	47	David Gibson
Strongyloididae	23	David Gibson
Subuluridae	16	David Gibson
Syngamidae	15	David Gibson
Syrphonematidae	1	David J. Hunt
Taeniidae	26	Rodney Bray
Telorchiidae	9	David Gibson
Tenuisentidae	1	David Gibson
Tetrabothriidae	11	Rodney Bray
Tetrameridae	26	David Gibson
Tetraonchidae	6	Rodney Bray
Thapariellidae	1	David Gibson
Thelastomatidae	28	David J. Hunt
Thelaziidae	14	David Gibson
Travassosinematidae	4	David J. Hunt
Triaenophoridae	10	Rodney Bray
Trichinellidae	6	David Gibson
Trichosomoididae	3	David Gibson
Trichostrongylidae	78	David Gibson
Trichuridae	21	David Gibson
Troglotrematidae	3	David Gibson
Typhlocoelidae	4	David Gibson
Zoogonidae	2	David Gibson

**Table 2.** Associated experts who have helped with various helminth groups.

GROUP or AREA	OTHER SPECIALIST(S)
Monogenea	Philip Harris
Cestoda	Boyko Georgiev
Cestoda	Tomaš Scholz
Digenea	Tor Bakke
Digenea	Teresa Pojmanska
Digenea	Katarzyna Niewiadomska
Digenea	Aneta Kostadinova
Digenea	Vasyl Tkach
Nematoda	Odile Bain [deceased]
Nematoda	Marie-Claude Durette-Desset
Nematoda	Lynda Gibbons
Nematoda	František Moravec
Nematoda	Annie Petter
Acanthocephala	Zlatka Dimitrova
Acanthocephala	Kurt Buchmann
Acanthocephala	Tellervo Valtonen
